# Difference in time and risk preferences: physicians and general population across genders

**DOI:** 10.1186/s13561-025-00653-4

**Published:** 2025-07-05

**Authors:** Shingo Kasahara, Hirotaka Kato, Rei Goto

**Affiliations:** 1 Graduate School of Business Administration, Keio University, 4-1-1 Hiyoshi, Yokohama, Kanagawa 223-8521 Japan; 2 School of Economics and Business Administration, Yokohama City University, 22–2 Seto, Kanazawa, Yokohama 236–0027 Japan

**Keywords:** Patient–physician concordance, Risk attitude, Risk aversion, Time preferences, Lottery choice, Behavioral experiments

## Abstract

**Background:**

The alignment of preferences between physicians and patients can cause variations in treatment decision-making, thereby affecting health outcomes. However, research on the differences in preferences between physicians and the general population is scarce. This study examines the risk and time preferences of physicians compared with those of the general population, exploring the influence of gender concordance on health outcomes and decision-making in healthcare.

**Methods:**

We conducted an online field experiment in October and November 2022 in Japan and analyzed the responses of 469 individuals, including physicians and the general population. The survey was stratified by age and gender to align with the demographics of physicians nationally. Participants’ preferences were measured across the health and monetary domains by using a modified multiple price list test format.

**Results:**

The findings revealed that physicians tended to be more risk-averse than the general population in the health and monetary domains, although no statistically significant differences were observed. Physicians were found to be statistically significantly future-oriented, particularly regarding their significant health or monetary gains. Furthermore, while the female general population was more risk-averse in both domains, a gender difference in the physician group was observed only in the monetary domain.

**Conclusion:**

The results affirm that preference differences between physicians and the general population exist in Japan and clarify the unique preference traits of female physicians.

**Supplementary Information:**

The online version contains supplementary material available at 10.1186/s13561-025-00653-4.

## Background

Patient–physician concordance has gained substantial attention in medicine and economics owing to its impact on health outcomes. Improved health outcomes and patient satisfaction have been observed when physicians and patients share the same gender or race [1–5]. Greenwood et al. [2] explored the effects of gender concordance on health outcomes in women with acute myocardial infarction. They revealed that when female patients are treated by female physicians, their health outcomes are notably better than when male physicians provide treatment. This highlights the potential influence of gender concordance on health outcomes. However, the mechanisms underlying the gender disparities between physicians and patients that result in different health outcomes remain under-explored.

The alignment of preferences between physicians and patients can cause variations in treatment decision-making, thereby affecting health outcomes. Risk and time preferences are fundamental components of decision-making because they determine how individuals value uncertain or delayed health gains [6–9]. Physicians are expected to discern patient preferences and help make optimal treatment selections; however, directly observing a patient’s time and risk preferences may be difficult. In reality, physicians often make treatment decisions based on their risk and time preferences instead of those of patients [10]. Earlier research reveals that risk-averse general practitioners in France would receive influenza vaccinations and recommend them to their patients [11]. When a physician’s risk or time preference diverges from that of the patient, treatment recommendations may deviate from the patient’s utility maximum, reducing adherence and ultimately worsening outcomes [12]. Moreover, considering that preferences may differ between genders [13], this disparity could explain the observed diminished performance when male physicians treat female patients.

To the best of our knowledge, research on the differences in risk and time preferences between physicians and the general population is limited. The framed field experiment conducted by Galizzi et al. [14] was an exception; it revealed that physicians place higher value on future outcomes and have similar risk preferences to patients in the healthcare domain. However, this study had several limitations. First, the results were from a single university hospital in Athens, making the sample too small to generalize the findings to other healthcare settings. Second, the study did not investigate variations in preferences among a group of physicians. Third, discrepancies in the questioning methods used by the physicians and patients were not addressed. Specifically, the authors suggested that patients completed the questionnaire with assistance, whereas physicians appeared to have answered it without such guidance. Given these concerns, unanswered questions exist regarding the differences in preferences between physicians and patients, specifically concerning the potential alignment between specific subgroups such as gender. Furthermore, given that numerous studies have shown that risk and time preferences are domain-specific [15–17], it is still uncertain whether the differences in preferences among physicians across domains are decisive. It is thus important to investigate whether preferences manifest differently in health and monetary domains.

This study aimed to analyze the differences in risk and time preferences between physicians and the general population. Additionally, we investigated the differences in risk and time preferences between male and female physicians and compare the results with those observed in the general population. We examined whether preferences vary across two domains (health and monetary). To this end, we conducted a nationwide online survey of 469 individuals in Japan, comprising both physicians and the general population, and analyzed the relationship between their characteristics and preferences.

## Material and methods

### Data

We conducted an online field experiment from October 31 to November 13, 2022, using two panels provided by Macromill, Inc., one of Japan’s leading Internet research companies. The physician panel included approximately 100,000 registered physicians, comprising approximately one-third of all physicians in Japan, making it one of the largest physician panels in the country. To ensure reliability, the research company verified the credentials of the panelists as qualified physicians and their basic information such as specialties and affiliated institutions. The general population panel included 1.3 million individuals from diverse backgrounds nationwide.

In this study, we stratified for the physician and general population samples by age and gender on the basis of the 2018 Statistics of Physicians, Dentists, and Pharmacists in Japan. We aimed not to obtain a fully representative sample of the general population but to minimize confounding due to demographic factors. By matching the distribution of age and gender, the study design facilitated a precise comparison of preferences between physicians and the general population.

Before the survey, we calculated the proportions for each gender and age category at five-year intervals based on the aforementioned 2018 statistics and set the intended number of participants for each category. Thereafter, invitations to participate in the survey were sent by Macromill Inc. to registered panel members based on their previously registered age and gender information. Invitations to panel members in a category stopped when the planned number of respondents was reached. As there were very few panel registrants over the age of 70 years, this age group was excluded from our survey. All participants provided informed consent electronically before beginning the survey. Before participation, they were presented with an online consent form explaining the voluntary nature of the study and their right to withdraw at any time. In our survey, we did not provide variable financial incentives to participants based on their answers. The data passed onto the researchers were anonymized. Macromill Inc. considered the number of invitations sent, the response rate among those invited, and the dropout rate during the survey to be confidential and not disclosed. The study was approved by the Ethics Review Committee of the Keio University Graduate School of Business Administration on January 18, 2022.

### Risk and time preferences

To assess the stated risk and time preferences, we adopted a questionnaire aligned with the format of the multiple price list (MPL) tests established by Holt and Laury [18], Tanaka et al. [19], and Galizzi et al. [14]. To measure risk preferences in the health domain, participants were presented with nine pairs of options for Treatments A and B and asked to choose either option. Each treatment had two possible outcomes in terms of the duration of the effect and probability of those outcomes. Table 1 presents the questionnaire items. Under Treatment A, there was a 10% chance of remaining in full health for 200 days and a 90% chance of remaining in full health for 160 days. Under Treatment B, there was a 10% chance of remaining in full health for 385 days and a 90% chance of remaining in full health for 10 days. Across the nine pairs, the number of full health days gained was constant; however, the associated probabilities varied.

**Table 1:** MPL test questionnaire to assess risk preferences in the health domain

ID	Treatment A				Treatment B			
	Probability	Days in full health	Probability	Days in full health	Probability	Days in full health	Probability	Days in full health
1	10%	200	90%	160	10%	385	90%	10
2	20%	200	80%	160	20%	385	80%	10
3	30%	200	70%	160	30%	385	70%	10
4	40%	200	60%	160	40%	385	60%	10
5	50%	200	50%	160	50%	385	50%	10
6	60%	200	40%	160	60%	385	40%	10
7	70%	200	30%	160	70%	385	30%	10
8	80%	200	20%	160	80%	385	20%	10
9	90%	200	10%	160	90%	385	10%	10

Participants were asked to choose between two treatment options, A and B, in each row of the questionnaire. Each treatment option had two possible outcomes associated with different probabilities. Treatment A offered a 10% chance of being in full health for 200 days and a 90% chance of being in full health for 160 days. By contrast, Treatment B offered a 10% chance of being in full health for 385 days and a 90% chance of being in full health for 10 days

The expected number of days in full health derived from each treatment option was calculated by multiplying each probability by its corresponding number of days and summing the two products. Treatment A offered a relatively high expected number of days in the initial few rows; however, in the fifth question, the expected outcome for Treatment B was higher than that for Treatment A. Therefore, a rational decision-maker would choose Treatment A initially, switch to Treatment B at the point at which its expected utility surpassed that of Treatment A, and continue to choose Treatment B thereafter. However, individuals did not simply choose the option with the highest expected value but rather selected the option that offered the greatest utility based on their risk preferences. Therefore, even rational individuals may not necessarily switch to the fifth line. A risk-averse individual would stick with the “safer” treatment (Treatment A) for longer or continue selecting Treatment A, whereas a risk-seeking individual would switch to the “riskier” treatment (Treatment B) sooner or consistently choose Treatment B throughout. Thus, the switching point from Treatment A to Treatment B indicated the risk preference of an individual, with a high value indicating high risk aversion. To measure monetary preferences, we asked participants to choose between Lotteries A and B instead of treatments and the amount of money received replaced full health days in the health domain questions. Please refer to Appendix B for the questionnaire.

For time preferences, participants were asked to respond to a series of six blocks, with each block containing five binary questions. In each block, choosing Treatment A resulted in a specific number of full health days, starting at a delayed date. Treatment B provided fewer full health days than Treatment A but became effective immediately. Within a block, the number of full health days offered by Treatment B started low and accumulated. Table 2 presents examples of specific questionnaire items. A rational decision-maker would start with Treatment A and switch to Treatment B midway through, continuing with Treatment B thereafter. Subsequently, such a decision-maker would never switch back to Treatment A. Alternatively, they may select Treatment A or B throughout a block. The point at which an individual switches from Treatment A to B, or continues with A, is determined by their time preference. Those who are future-oriented delay switching to Treatment B for an immediate but short duration. Thus, the switching point from Treatment A to B indicates the time preference of the individual, with a later switching point showing a significant emphasis on the future. Consistent with the structure adopted in earlier study [14], the differences in the composition of the six blocks were as follows: the delay in the onset of Treatment A’s effect was short (1 week) in Blocks 1 and 4, intermediate (1 month) in Blocks 2 and 5, and long (3 months) in Blocks 3 and 6. The number of full health days was relatively low (60–360 days) in Blocks 1–3 and relatively high in Blocks 4–6 (150–900 days). For monetary preferences, the same modifications were made to the risk preference questionnaire items. We adopted the same pricing structure and day intervals as those employed by Galizzi et al. [14] in their questionnaire (see Appendix B for the questionnaire).

**Table 2:** MPL test questionnaire to assess time preferences in the health domain (excerpt)

Block	ID	Number of days in full health	
		Treatment A	Treatment B
1	1	360 days starting in 1 week	60 days starting today
1	2	360 days starting in 1 week	120 days starting today
1	3	360 days starting in 1 week	180 days starting today
1	4	360 days starting in 1 week	240 days starting today
1	5	360 days starting in 1 week	300 days starting today

Participants were asked to choose between two treatment options, A and B, in each row of the questionnaire. Choosing Treatment A would result in a specific number of full health days, starting at a delayed date. Choosing Treatment B would provide fewer full health days than Treatment A but would be effective immediately

Additionally, the questionnaire collected information on participants’ socioeconomic characteristics such as gender, age, and income. Questions other than those used to assess preferences were obtained from the Japan Preference Parameter Study conducted by the Institute of Social and Economic Research at Osaka University. The specific questions are presented in Appendix B.

### Analytical steps

First, we compared the preference indicators (switching points from Treatment A to B) between the physician and general population groups. The results of the Shapiro–Wilk tests for normality rejected the null hypothesis that the switching points were normally distributed in each group; therefore, we used the non-parametric Mann–Whitney U test to examine the differences in the values of the risk and time preference indicators for each block between physicians and the general population.

Subsequently, gender differences in the physician group were determined and compared with those in the general population. To this end, we compared the risk and time preference indicators across four groups (male general population, male physicians, female general population, and female physicians) using the ordinary least squares method, while controlling for age and income. We determined whether there was a significant difference at the 5% level. The analysis was conducted using STATA version 17.

## Results

Table 3 presents the descriptive statistics. We obtained 226 and 243 responses from the physician and general population panels, respectively. The average age of physicians was 47.2 years, whereas that of the general population was 45.9 years. The proportion of women was 23% in the physician group and 28% in the general population group. The age and gender distributions did not significantly differ between the physician and general population groups, as indicated by a chi-square test (*p* = 0.99). According to the 2018 statistics used, the average age of Japanese physicians was 49.9 years and 21.9% were female, indicating that we recruited participants closely matching the age and gender distribution of physicians nationwide. The number of participants by age and gender is shown in Appendix A. Physicians had high income levels as expected and most possessed a bachelor’s degree (including Bachelor of Medicine) or higher. By contrast, only 57.2% of the general population reported having attained a bachelor’s degree or higher.

**Table 3:** Summary statistics of the physician and general population groups

	Physicians					General population				
	n	Mean/Prop	SD	Max	Min	n	Mean/Prop	SD	Max	Min
age	226	47.2	12.3	69	25	243	45.9	12.2	67	27
female	226	0.23	0.42	1	0	243	0.28	0.45	1	0
annual income (Japanese yen)	226	16,334,770	24,313,021	300,000,000	5	243	3,446,493	5,085,037	50,000,000	0
bachelor’s degree or higher	226	1.00	0	1	0	243	0.57	0.49	1	0
risk_health	197	7.82	2.52	10	1	202	7.59	2.84	10	1
risk_fin	183	6.61	2.75	10	1	183	6.74	2.99	10	1
time_health_b1	213	5.61	1.08	6	1	213	5.32	1.52	6	1
time_health_b2	215	5.27	1.40	6	1	219	4.88	1.80	6	1
time_health_b3	212	4.91	1.53	6	1	219	4.47	1.90	6	1
time_health_b4	212	5.51	1.30	6	1	217	4.98	1.82	6	1
time_health_b5	211	5.32	1.40	6	1	219	4.86	1.79	6	1
time_health_b6	213	5.02	1.63	6	1	219	4.55	1.89	6	1
time_fin_b1	209	5.52	1.21	6	1	215	5.43	1.34	6	1
time_fin_b2	210	5.34	1.36	6	1	221	5.01	1.70	6	1
time_fin_b3	212	5.25	1.41	6	1	222	4.76	1.82	6	1
time_fin_b4	215	5.57	1.25	6	1	223	5.36	1.47	6	1
time_fin_b5	212	5.46	1.36	6	1	220	5.11	1.64	6	1
time_fin_b6	213	5.36	1.37	6	1	225	4.93	1.74	6	1

The sample size (n) for the risk and time preference indicators reflects the count after excluding responses that included at least one switching back event

Most participants responded to the questions on the preference indicators using their rational judgment. For risk preferences, the average switching points for both domains were greater than five, the point at which the expected value for Treatment A was greater than that for Treatment B. In other words, both physicians and the general population exhibited a tendency toward risk aversion. However, for the risk preference responses, 12.8% of physicians in the health domain and 19.0% in the monetary domain switched back from Treatment B to Treatment A at least once in the series of questions. Similarly, 16.9% of the general population in the health domain and 24.7% in the monetary domain switched back from Treatment B to Treatment A. Regarding time preferences, fewer than 10% of physicians and the general population switched back, with no significant differences between the health and monetary domains. These responses were excluded from the primary analyses.

In the primary analyses, we investigated whether there were differences in the preference indicators between physicians and the general population. Table 4 presents the results. When comparing the average values, physicians had high switching points in all the columns for both the health and the monetary domains, suggesting that they are more risk-averse and future-oriented than the general population. Next, we verified the differences in each preference indicator between the groups using a non-parametric test (Mann–Whitney U test). There was no statistically significant difference in risk preferences between the groups. For time preferences, when testing for significant differences in the preference indicators by block, statistical significance was reached in the health domain (Blocks B4–B6) and monetary domain (Blocks B3–B6). This suggests that physicians were considerably patient for expected future benefits, particularly in blocks with large gains in days in full health or monetary benefits.

**Table 4:** Differences in risk and time preferences between physicians and the general population

	Risk	Block 1	Block 2	Block 3	Block 4	Block 5	Block 6
Health domain							
Observations of physicians	197	213	215	212	212	211	213
Observations of the general population	202	213	219	219	217	219	219
Mean switching point: physicians	7.82	5.61	5.27	4.91	5.51	5.32	5.02
Mean switching point: general population	7.59	5.32	4.88	4.47	4.98	4.86	4.55
Z-statistic	− 0.31	− 1.556	− 1.863	− 1.936	− 3.448	− 2.646	− 2.639
*P*-value	0.75	0.120	0.062	0.053	0.001	0.008	0.008
Monetary domain							
Observations of physicians	183	209	210	212	215	212	213
Observations of the general population	183	215	221	222	223	220	225
Mean switching point: physicians	6.61	5.52	5.34	5.25	5.57	5.46	5.36
Mean switching point: general population	6.74	5.43	5.01	4.76	5.36	5.11	4.93
Z-statistic	0.621	− 0.150	− 1.779	− 2.360	− 1.746	− 2.798	− 2.373
*P*-value	0.53	0.882	0.075	0.018	0.081	0.005	0.018

The z-statistics and *p*-values show the results of the Mann–Whitney U tests, where the null hypothesis is that the switching points are not statistically significantly different between physicians and the general population

Subsequently, we conducted a regression analysis using the ordinary least squares method, controlling for age and income, with the male general population as the reference to compare the preference indicators across the four groups (male general population, male physicians, female general population, and female physicians). Tables 5 and 6 presents the results of this analysis. Panel A (B) shows the regression results for the health (monetary) domain preferences. For risk preferences, all the groups were more risk-averse than the general male population in both the health and the monetary domains. However, there was no statistically significant difference in health-related risk preferences among the three groups and the general male population. For monetary risk preferences, the only significant difference, at the 5% level, was observed in the female physician group.

**Table 5:** Comparison of the four groups. Panel A: health domain

	Risk	Block 1	Block 2	Block 3	Block 4	Block 5	Block 6
General male population							
(reference)							
Male physicians	0.418	0.288	0.385*	0.502*	0.508**	0.490*	0.565**
	(− 1.26)	(− 1.81)	(− 2.00)	(− 2.41)	(− 2.68)	(− 2.51)	(− 2.62)
General female population	0.698	0.00917	− 0.0439	− 0.0275	0.257	0.189	0.0508
	(− 1.43)	(− 0.04)	(− 0.16)	(− 0.09)	(− 0.93)	(− 0.67)	(− 0.16)
Female physicians	0.452	0.27	0.34	0.278	0.873**	0.673*	0.237
	(− 0.97)	(− 1.22)	(− 1.25)	(− 0.94)	(− 3.26)	(− 2.44)	(− 0.79)
Age	− 0.134*	0.0078	0.0206	0.0225	0.0383	0.0583	0.02
	(− 2.31)	(− 0.28)	(− 0.62)	(− 0.63)	(− 1.17)	(− 1.73)	(− 0.54)
Income	− 0.0168	0.00411	0.00802	0.0135	0.0103	0.00676	0.0212
	(− 0.38)	(− 0.19)	(− 0.31)	(− 0.48)	(− 0.4)	(− 0.26)	(− 0.75)
N	378	404	411	408	406	408	410
R-squared	0.0253	0.0126	0.0170	0.0231	0.0392	0.0314	0.0277

t-statistics in parentheses. * *p* < 0.05, ** *p* < 0.01, *** *p* < 0.001

**Table 6:** Comparison of the four groups. Panel B: monetary domain

	Risk	Block 1	Block 2	Block 3	Block 4	Block 5	Block 6
General male population							
(reference)							
Male physicians	0.236	− 0.0613	0.283	0.464*	0.208	0.276	0.303
	(− 0.64)	(− 0.42)	(− 1.58)	(− 2.45)	(− 1.34)	(− 1.57)	(− 1.64)
General female population	0.772	0.246	0.531*	0.232	0.487*	0.406	0.0917
	(− 1.41)	(− 1.16)	(− 2.04)	(− 0.84)	(− 2.14)	(− 1.58)	(− 0.35)
Female physicians	1.071*	0.195	0.452	0.643*	0.175	0.535*	0.555*
	(− 2.11)	(− 0.95)	(− 1.78)	(− 2.41)	(− 0.80)	(− 2.14)	(− 2.12)
Age	0.0234	− 0.0501*	− 0.0527	− 0.0152	− 0.0287	− 0.0241	− 0.0188
	(− 0.36)	(− 1.97)	(− 1.70)	(− 0.46)	(− 1.07)	(− 0.79)	(− 0.59)
Income	− 0.0676	0.0187	0.0165	− 0.0174	0.0129	0.0216	0.0185
	(− 1.37)	(− 0.96)	(− 0.70)	(− 0.69)	(− 0.62)	(− 0.91)	(− 0.75)
N	348	401	409	411	415	410	415
R-squared	0.0213	0.0213	0.0290	0.0222	0.0178	0.0232	0.0212

t-statistics in parentheses. * *p* < 0.05, ** *p* < 0.01, *** *p* < 0.001. Income is added into all the regression models as the natural logarithm of annual income (Japanese yen)

**Fig. 1:**
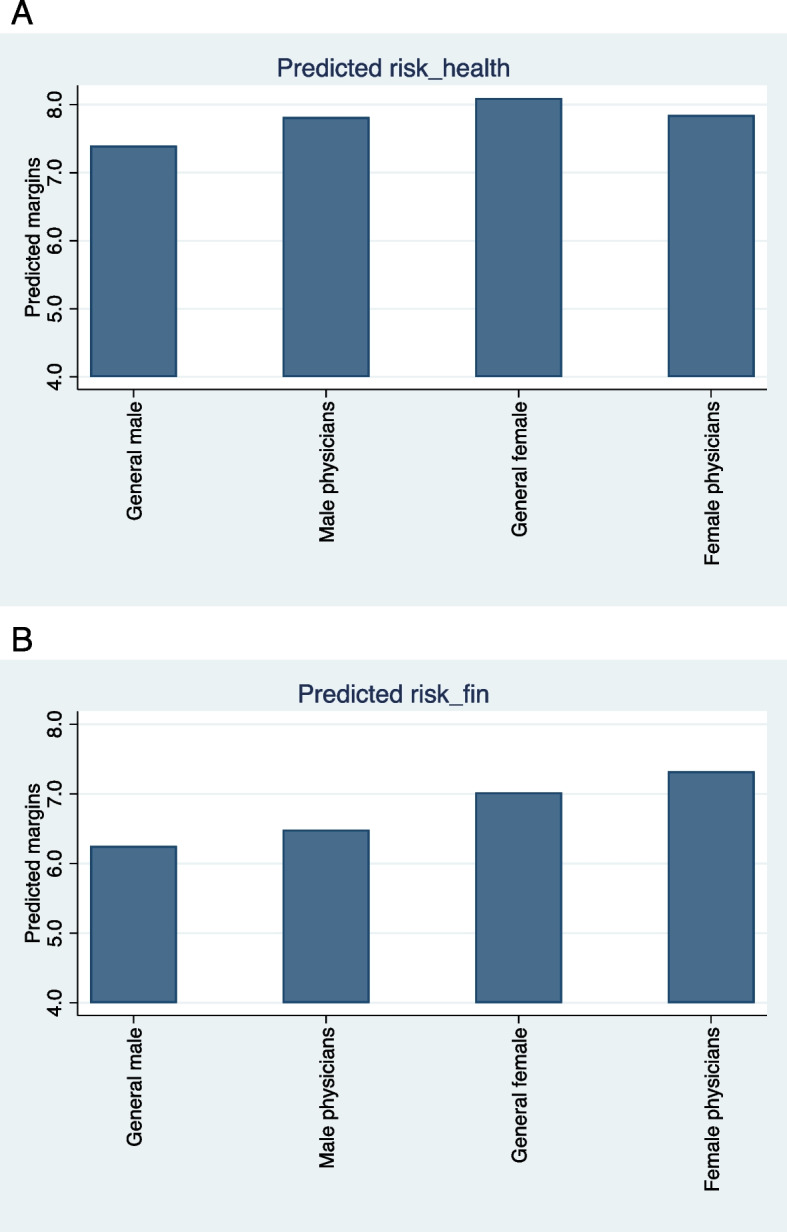
Predictive margins by domain and group. Panel **A**: Health Domain. Panel **B**: Monetary Domain

For time preferences, all three groups had higher indicators of future orientation in all the blocks than the general male population in both the health and the monetary domains. In the health domain, male physicians were statistically more future-oriented than the male general population in the five blocks. A similar trend was observed in the female physician group; however, significant differences were found only in two blocks. The general female population did not differ significantly from the general male population. In the monetary domain, significant differences were found in one block for male physicians, two blocks for the general female population, and three blocks for female physicians. For visual presentation, the risk and time preference indicators for each domain are displayed in bar-chart panels (see Fig. 1).

## Discussion

Based on the literature showing that gender concordance between patients and physicians affects health outcomes, our study explored the possible underlying pathways of the differences in preferences between physicians and the general population as well as between genders. Physicians tended to be more risk-averse than the general population in both health and monetary domains, although no statistically significant differences were observed. Furthermore, physicians were future-oriented in both domains. From the perspective of gender differences, a novel aspect of this study is that while the general female population was considerably risk-averse in both domains, a gender difference in the physician group was observed only in the monetary domain.

Our results from comparing the physician group with the general population are consistent with those of Galizzi et al. [14]. However, unlike their study that surveyed physicians and patients in an outpatient setting at a single university hospital in Athens, our study collected samples from physicians and the general population across Japan. These findings demonstrate that insights from prior research hold for a relatively broad demographic of physicians and the general population in a different country. The pronounced future-oriented tendency in blocks with large gains in full health days or monetary benefits suggests that the more serious the health outcomes associated with a decision, the stronger the physician’s future-oriented tendency.

Concerning preference differences by gender, while female physicians showed a tendency toward risk aversion in the monetary domain, no gender differences were observed in the health domain. Considering previous findings that women are generally more risk-averse [13], understanding this divergence is crucial for comprehending the preference differences between male and female physicians and the general population. As the two groups differ in profession, the influence of professional education and training that physicians receive could cause female physicians to make different health-related decisions than the general female population. Male physicians occupy leadership positions in the field of medical education in many developed countries. In the United States, there are 30,464 female medical school faculties and 60,609 male counterparts, of which 3,623 women (11.9%) and 17,354 men (28.6%) have full-professor appointments [20]. In Japan, this tendency is stronger, as all 82 medical schools have male deans and hospital directors [21]. Physicians’ workplace culture is hierarchical, with senior physicians imparting the “correct” decision-making methods to junior physicians through medical education and postgraduate training. Female medical students and physicians in Japan may be implicitly or explicitly encouraged to behave like male instructors or superiors through medical education and professional training. This potentially leads to the disappearance of gender differences in health-related time preferences among physicians.

Beyond professional socialization, non-work burdens offer an additional explanation. Cross-national sociological research classifies Japan into a “pro-work conservatism” cluster, where women shoulder a disproportionate share of domestic labor and enjoy only limited socially acceptable ways of combining employment and child-rearing [22]. Recent national data show that female physicians with children work markedly fewer hours and undertake fewer night shifts than male physicians (≥ 60 h/week: 14.5% vs. 35.5% [23]. International evidence points in the same direction: using Austrian administrative data, Pruckner et al. report that female physicians worldwide tend to reduce hours once family responsibilities intensify [24]. Heavy dual-role responsibilities are associated with larger health losses for women, especially when household tasks are not shared with a spouse [25]. Such responsibilities may heighten risk aversion in the monetary domain, while having an ambiguous impact on health-related choices, potentially accounting for the domain-specific pattern observed in our results. We emphasize that this mechanism remains speculative and should be tested in future research.

Furthermore, a significant difference was observed in the proportion of individuals with a bachelor’s degree or higher between physicians and the general population (97.3% vs. 57.2%). Entry into Japanese medical schools now requires increasingly high academic achievement [26], implying that the true educational gap between the two groups may exceed what is captured by degree status alone. Because higher educational attainment has been reported to be associated with greater patience [27–29], we re-estimated all the models controlling for educational background; the findings were virtually unchanged (see Appendix C).

The primary limitation of this study was the high response rate with at least one switching back event. Approximately 18% of the risk preference responses and 6% of the time preference responses included switching back from Treatment B to Treatment A at some point within the series of questions. The MPL test design may have contributed to the cognitive burden on participants, leading to a high switching back rate. However, the switching back rate in our study is significantly higher than that 4 of 300 patients (1.3%) in Galizzi et al.’s experiment [14]. The questionnaire instructions in our study closely matched those of Galizzi et al.’s [14], ruling it out as a primary source of the observed differing outcomes. This difference may have occurred because in Galizzi et al.’s [14] experiment, survey staff actively helped participants, which may have improved participants’ understanding of the questionnaire. By contrast, participants completed our survey alone, which could partly explain the higher switching back rate. As a sensitivity analysis, we retained only respondents who switched back and defined their switching points by the first row in which they switched from Treatment A to Treatment B; re-estimating all the models with this rule produced essentially the same results (see Appendix D). We also analyzed whether the risk preference of respondents who exhibited high rates of switching back had distinct characteristics. No significant differences were observed in gender, age, income, or educational background between the group that switched back at least once and the group that never switched back.

Another limitation concerns the representativeness of our samples. First, although the physician sample was stratified by age and gender to mirror the national workforce, recruiting through an online panel may still over-represent doctors who are more accustomed to using the internet, so our data may not fully capture the profession’s diversity. Second, because we adopted the same gender ratio for the general population, women accounted for only 28% of respondents, meaning this group cannot be considered nationally representative either. Finally, because individual preferences are partly shaped by cultural context, the extent to which our findings can be generalized outside of Japan remains uncertain; accumulating evidence from studies conducted in other countries will be essential.

Finally, we interpreted risk and time preferences as relatively stable traits [30, 31]; however, recent evidence shows that exogenous shocks such as financial crises, trauma from violence, and natural disasters can shift individuals’ preferences [32–35]. A longitudinal design that tracks the same physicians (or medical students) before and after such shocks would therefore provide stronger causal insights into how clinical experience and external events shape preference formation.

## Conclusion

This study investigated the risk and time preferences of physicians and the general population through an online field experiment. An MPL test was employed to measure the risk and time preferences. Physicians tended to be risk-averse, although no statistically significant differences were found compared with the general population. Physicians were also significantly future-oriented in both health and monetary domains. The general female population was more risk-averse than their male counterparts in both domains, whereas gender differences in the physician group were observed only in the monetary domain. This suggests that the professional education and training of physicians may affect their health-related decisions.

## Supplementary Information

Supplementary Material 1.

## Data Availability

Data can be made available upon reasonable request to the corresponding author.
