# Supplementary material for: Difference in time and risk preferences: physicians and general population across genders
Source: Health Econ Rev. 2025 Jul 5;15:56. doi: 10.1186/s13561-025-00653-4 (PMC12228394; doi:10.1186/s13561-025-00653-4)
Supplement: Supplementary file 1 — Supplementary Material 1. [file 13561_2025_653_MOESM1_ESM.docx]

**Difference in time and risk preferences: Physicians and general population across genders**

**Appendix A: Age distribution of the physicians and general population by gender**

| Gender | Age (years) | Physicians (n = 226) | |  | General population (n = 243) | |  | Numbers of physicians in 2018 |
| --- | --- | --- | --- | --- | --- | --- | --- | --- |
|  |  | N | (%) |  | N | (%) |  | (%) |
| Male | 25–29 | 16 | 7.1 |  | 14 | 5.8 |  | 6.4 |
|  | 30–34 | 19 | 8.4 |  | 23 | 9.5 |  | 7.8 |
|  | 35–39 | 19 | 8.4 |  | 19 | 7.8 |  | 7.8 |
|  | 40–44 | 20 | 8.8 |  | 19 | 7.8 |  | 8.3 |
|  | 45–49 | 21 | 9.3 |  | 20 | 8.2 |  | 9.2 |
|  | 50–54 | 22 | 9.7 |  | 21 | 8.6 |  | 9.6 |
|  | 55–59 | 20 | 8.8 |  | 23 | 9.5 |  | 10.4 |
|  | 60–64 | 21 | 9.3 |  | 21 | 8.6 |  | 9.8 |
|  | 65–69 | 16 | 7.1 |  | 16 | 6.6 |  | 7.3 |
| Female | 25–29 | 8 | 3.5 |  | 9 | 3.7 |  | 3.5 |
|  | 30–34 | 8 | 3.5 |  | 9 | 3.7 |  | 3.6 |
|  | 35–39 | 8 | 3.5 |  | 9 | 3.7 |  | 3.5 |
|  | 40–44 | 8 | 3.5 |  | 9 | 3.7 |  | 3.5 |
|  | 45–49 | 7 | 3.1 |  | 9 | 3.7 |  | 2.9 |
|  | 50–54 | 6 | 2.7 |  | 7 | 2.9 |  | 2.3 |
|  | 55–59 | 5 | 2.2 |  | 6 | 2.5 |  | 1.8 |
|  | 60–64 | 2 | 0.9 |  | 5 | 2.1 |  | 1.4 |
|  | 65–69 | 0 | 0 |  | 4 | 1.6 |  | 0.8 |

**Appendix B: Questionnaire of the participants’ preferences in the health and monetary domains**

This survey is conducted by the Goto Laboratory, Graduate School of Business Administration, Keio University to determine the time and risk preferences of Japanese physicians in the health and monetary domains. If you participate in the study, your personal information will be handled appropriately and your privacy will be protected. The results of the study may be published at conferences or in research articles but will not contain any personally identifiable information. If you feel uneasy or uncomfortable about any of the questions in the survey, you may end the survey anytime.

**Survey**

1. How would you describe your current health status?

a. Excellent

b. Good

c. Fair

d. Poor

e. Bad

2. Do you have a spouse?

a. Currently have a spouse (a husband or wife, including common-law marriage)

b. Had a spouse in the past and no longer have one (separated)

c. Had a spouse in the past and no longer have one (died)

d. Never had a spouse

3. Do you have children?

a. Yes

b. No

4. What is your smoking habit?

a. I smoke more than 41 cigarettes a day

b. I smoke about 31–40 cigarettes a day

c. I smoke about 21–30 cigarettes a day

d. I smoke about 11–20 cigarettes a day

e. I smoke about 6–10 cigarettes a day

f. I smoke about 1–5 cigarettes a day

g. I occasionally smoke

h. I do not smoke but used to smoke almost daily in the past

i. I do not smoke but used to occasionally smoke in the past

j. Never smoked

5. What is your drinking habit?

a. I drink almost every day, more than 5 cans of beer (350 ml)

b. I drink almost every day, about 3 cans of beer (350 ml)

c. I drink almost every day, about 1 can of beer (350 ml)

d. I occasionally drink (several times a week)

e. I hardly drink (a few times a month or less)

f. I don’t drink at all.

6. What is your exercise habit?

a. Almost every day

b. 2–4 times a week

c. About once a week

d. About once a month

e. Rarely

7. Tell us how you perceive your personality. Are you a person who is willing to take risks or a person who avoids risks? Assuming that 0 means "not at all willing to take risks" and 10 means "willing to take risks," where would you place yourself between 0 and 10?

a. With respect to the management of your personal assets

b. With respect to behavior related to your health

8. Each of the following rows is a pair of hypothetical lotteries. and forFor each row, choose your favorite of the two lotteries, A or B. Lottery A will give you either 20,000 yen or 16,000 yen with some probabilities that change in each row. Lottery B give you either 38,500 yen or 1,000 yen with some probabilities that change in each row.

For example, in the first line, Lottery A has a 10% chance of winning 20,000 yen and a 90% chance of winning 16,000 yen, while Lottery B has a 10% chance of winning 38,500 yen and a 90% chance of winning 1,000 yen.

There is no right or wrong answer. Please answer based on your personal choice.

| ID | Lottery A | | | | Lottery B | | | |
| --- | --- | --- | --- | --- | --- | --- | --- | --- |
|  | Probability | ¥ | Probability | ¥ | Probability | ¥ | Probability | ¥ |
| 1 | 10% | 20,000 | 90% | 16,000 | 10% | 38,500 | 90% | 1,000 |
| 2 | 20% | 20,000 | 80% | 16,000 | 20% | 38,500 | 80% | 1,000 |
| 3 | 30% | 20,000 | 70% | 16,000 | 30% | 38,500 | 70% | 1,000 |
| 4 | 40% | 20,000 | 60% | 16,000 | 40% | 38,500 | 60% | 1,000 |
| 5 | 50% | 20,000 | 50% | 16,000 | 50% | 38,500 | 50% | 1,000 |
| 6 | 60% | 20,000 | 40% | 16,000 | 60% | 38,500 | 40% | 1,000 |
| 7 | 70% | 20,000 | 30% | 16,000 | 70% | 38,500 | 30% | 1,000 |
| 8 | 80% | 20,000 | 20% | 16,000 | 80% | 38,500 | 20% | 1,000 |
| 9 | 90% | 20,000 | 10% | 16,000 | 90% | 38,500 | 10% | 1,000 |

9. Each of the following rows is a pair of hypothetical choices. For each row, choose your favorite of the two options. Under both options, assume that you will receive a certain amount of money. Payment for Choice A will be made at a later date; payment for Choice B will be made today.

There is no right or wrong answer. Please answer based on your personal choice.

| ID | Option A | Option B |
| --- | --- | --- |
| 1 | Receive 36,000 yen in 1 week | Receive 6,000 yen today |
| 2 | Receive 36,000 yen in 1 week | Receive 12,000 yen today |
| 3 | Receive 36,000 yen in 1 week | Receive 18,000 yen today |
| 4 | Receive 36,000 yen in 1 week | Receive 24,000 yen today |
| 5 | Receive 36,000 yen in 1 week | Receive 30,000 yen today |
|  |  |  |
| 6 | Receive 36,000 yen in 1 month | Receive 6,000 yen today |
| 7 | Receive 36,000 yen in 1 month | Receive 12,000 yen today |
| 8 | Receive 36,000 yen in 1 month | Receive 18,000 yen today |
| 9 | Receive 36,000 yen in 1 month | Receive 24,000 yen today |
| 10 | Receive 36,000 yen in 1 month | Receive 30,000 yen today |
|  |  |  |
| 11 | Receive 36,000 yen in 3 months | Receive 6,000 yen today |
| 12 | Receive 36,000 yen in 3 months | Receive 12,000 yen today |
| 13 | Receive 36,000 yen in 3 months | Receive 18,000 yen today |
| 14 | Receive 36,000 yen in 3 months | Receive 24,000 yen today |
| 15 | Receive 36,000 yen in 3 months | Receive 30,000 yen today |
|  |  |  |
| 16 | Receive 90,000 yen in 1 week | Receive 15,000 yen today |
| 17 | Receive 90,000 yen in 1 week | Receive 30,000 yen today |
| 18 | Receive 90,000 yen in 1 week | Receive 45,000 yen today |
| 19 | Receive 90,000 yen in 1 week | Receive 60,000 yen today |
| 20 | Receive 90,000 yen in 1 week | Receive 75,000 yen today |
|  |  |  |
| 21 | Receive 90,000 yen in 1 month | Receive 15,000 yen today |
| 22 | Receive 90,000 yen in 1 month | Receive 30,000 yen today |
| 23 | Receive 90,000 yen in 1 month | Receive 45,000 yen today |
| 24 | Receive 90,000 yen in 1 month | Receive 60,000 yen today |
| 25 | Receive 90,000 yen in 1 month | Receive 75,000 yen today |
|  |  |  |
| 26 | Receive 90,000 yen in 3 months | Receive 15,000 yen today |
| 27 | Receive 90,000 yen in 3 months | Receive 30,000 yen today |
| 28 | Receive 90,000 yen in 3 months | Receive 45,000 yen today |
| 29 | Receive 90,000 yen in 3 months | Receive 60,000 yen today |
| 30 | Receive 90,000 yen in 3 months | Receive 75,000 yen today |

10. Consider the following hypothetical scenario. Suppose you have to choose between two treatments, A and B. Each treatment has two possible outcomes in terms of how long the effect will last and you know the probabilities. Whichever treatment you choose, you will remain in full health during the duration of its effect. When the effects of the treatment wear off, you will return to the initial state of health in which you were before you started treatment and no further treatment will be available.

For example, under Treatment A, you have a 10% chance of staying in full health for 200 days and a 90% chance of staying in full health for 160 days. Under Treatment B, you have a 10% chance of staying in full health for 385 days and a 90% chance of staying in full health for 10 days.

There is no right or wrong answer. Please answer based on your personal choice.

|  | Treatment A | | | | Treatment B | | | |
| --- | --- | --- | --- | --- | --- | --- | --- | --- |
| ID | Probability | Days in full health | Probability | Days in full health | Probability | Days in full health | Probability | Days in full health |
| 1 | 10% | 200 | 90% | 160 | 10% | 385 | 90% | 10 |
| 2 | 20% | 200 | 80% | 160 | 20% | 385 | 80% | 10 |
| 3 | 30% | 200 | 70% | 160 | 30% | 385 | 70% | 10 |
| 4 | 40% | 200 | 60% | 160 | 40% | 385 | 60% | 10 |
| 5 | 50% | 200 | 50% | 160 | 50% | 385 | 50% | 10 |
| 6 | 60% | 200 | 40% | 160 | 60% | 385 | 40% | 10 |
| 7 | 70% | 200 | 30% | 160 | 70% | 385 | 30% | 10 |
| 8 | 80% | 200 | 20% | 160 | 80% | 385 | 20% | 10 |
| 9 | 90% | 200 | 10% | 160 | 90% | 385 | 10% | 10 |

11. Consider the following hypothetical scenario. Suppose you suffer from a medical condition that affects your health. You have a choice between two treatments, A and B. Treatment A is available at a later date, and Treatment B is available today. Regardless of the treatment start date, once you begin treatment, its effects will last for the number of days listed in each option. For example, with the first combination, Treatment A allows you to stay in full health for 360 days starting one week from now, while Treatment B allows you to stay in full health for 60 days starting today. At the end of the treatment, you will return to the state of health in which you were before you started treatment, and no further treatment will be available. There is no other difference between Treatments A and B.

There is no right or wrong answer. Please answer based on your personal choice.

| ID | Treatment A | Treatment B |
| --- | --- | --- |
| 1 | 360 days in full health starting in 1 week | 60 days in full health starting today |
| 2 | 360 days in full health starting in 1 week | 120 days in full health starting today |
| 3 | 360 days in full health starting in 1 week | 180 days in full health starting today |
| 4 | 360 days in full health starting in 1 week | 240 days in full health starting today |
| 5 | 360 days in full health starting in 1 week | 300 days in full health starting today |
|  |  |  |
| 6 | 360 days in full health starting in 1 week | 60 days in full health starting today |
| 7 | 360 days in full health starting in 1 week | 120 days in full health starting today |
| 8 | 360 days in full health starting in 1 week | 180 days in full health starting today |
| 9 | 360 days in full health starting in 1 week | 240 days in full health starting today |
| 10 | 360 days in full health starting in 1 week | 300 days in full health starting today |
|  |  |  |
| 11 | 360 days in full health starting in 1 week | 60 days in full health starting today |
| 12 | 360 days in full health starting in 1 week | 120 days in full health starting today |
| 13 | 360 days in full health starting in 1 week | 180 days in full health starting today |
| 14 | 360 days in full health starting in 1 week | 240 days in full health starting today |
| 15 | 360 days in full health starting in 1 week | 300 days in full health starting today |
|  |  |  |
| 16 | 900 days in full health starting in 1 week | 150 days in full health starting today |
| 17 | 900 days in full health starting in 1 week | 300 days in full health starting today |
| 18 | 900 days in full health starting in 1 week | 450 days in full health starting today |
| 19 | 900 days in full health starting in 1 week | 600 days in full health starting today |
| 20 | 900 days in full health starting in 1 week | 750 days in full health starting today |
|  |  |  |
| 21 | 900 days in full health starting in 1 week | 150 days in full health starting today |
| 22 | 900 days in full health starting in 1 week | 300 days in full health starting today |
| 23 | 900 days in full health starting in 1 week | 450 days in full health starting today |
| 24 | 900 days in full health starting in 1 week | 600 days in full health starting today |
| 25 | 900 days in full health starting in 1 week | 750 days in full health starting today |
|  |  |  |
| 26 | 900 days in full health starting in 1 week | 150 days in full health starting today |
| 27 | 900 days in full health starting in 1 week | 300 days in full health starting today |
| 28 | 900 days in full health starting in 1 week | 450 days in full health starting today |
| 29 | 900 days in full health starting in 1 week | 600 days in full health starting today |
| 30 | 900 days in full health starting in 1 week | 750 days in full health starting today |

12. How much was your total personal income in the last year? *Please tell us the amount, including taxes.

13. Have you or any of your relatives been hospitalized in the past 3 years? (Include hospitalization for delivery. Do not include health checkups. If more than one option applies, choose the option with the earliest letter in alphabetical order)

| Relationship | Options | |
| --- | --- | --- |
| Yourself | Yes | No |
| Your spouse | Yes | No |
| Your parent or child | Yes | No |
| Your grandparents or siblings | Yes | No |

14. What is the highest level of education you have completed?

a. Bachelor (including Bachelor of Medicine)

b. Master

c. PhD

d. Other

15. How many years of experience do you have as a physician?

16. In which university did you complete your medical studies when you obtained your medical license?

17. Upon your admission to medical school, were you selected through a regional quota system? (Note: The regional quota system prioritizes the selection of students committed to serving in local medical practices, including those who are local residents and those who intend to work at a university and its affiliated hospitals. This selection method does not require scholarship loans.)

a. Yes

b. No

18. What is your primary method of paying for college tuition for your medical program?

a. Support from family

b. Scholarship (benefit type)

c. Scholarship (loan type)

d. Own expense

19. What was the occupation of the primary income earner in your family when you grew up? (e.g., if your family consisted of father, mother, and you, and your father was a physician and your mother was a housewife, please choose “physician”)

a. Physician

b. Dentist

c. Other healthcare professional

d. Non-healthcare professional

e. Unemployed

20. What is your work status?

a. Founder or representative of a legal entity

b. Employee

c. Unemployed

21. Are you affiliated with a specific department (medical office) at a specific university?

a. Yes

b. No

22. What is the average annual number of patients for which you have provided end-of-life care over the past 3 years?

23. What is your monthly average number of holiday workdays and night duty shifts over the last 3 years?

24. What is your primary specialty? (choose one from the following list)

a. General Internal Medicine

b. Cardiology

c. Nephrology

d. Gastroenterology

e. Respiratory Medicine

f. Allergology

g. Diabetology and Metabolism

h. Rheumatology

i. Hematology

j. Neurology

k. Psychiatry

l. Surgery

m. Orthopedics

n. Neurosurgery

o. Obstetrics and Gynecology

p. Dermatology

q. Urology

r. Otolaryngology

s. Pediatrics

t. Ophthalmology

u. Other

25. What is the management style of the facility with which you are affiliated?

a. University hospital

b. National or public hospital

c. Other general hospital

d. Private practice/clinic

e. Other

26. How many beds does your facility have?

a. 0

b. 1–19

c. 20–99

d. 100–199

e. 200–299

f. 300–399

g. 400–499

h. More than 500

27. What is the location of the facility in which you are primarily engaged?

a. (Select prefecture)

**Appendix C: Comparison of the four groups (with educational background controlled for)**

Panel A: Health domain

|  | Risk | Block 1 | Block 2 | Block 3 | Block 4 | Block 5 | Block 6 |
| --- | --- | --- | --- | --- | --- | --- | --- |
|  |  |  |  |  |  |  |  |
| Male general population |  |  |  |  |  |  |  |
| (reference) |  |  |  |  |  |  |  |
| Male physicians | 0.174 | 0.198 | 0.210 | 0.195 | 0.371* | 0.246 | 0.297 |
|  | (−1.200) | (−1.070) | (−1.310) | (−1.310) | (−2.110) | (−1.490) | (−1.920) |
| Female general population | 0.289 | 0.047 | 0.034 | 0.116 | 0.287 | 0.181 | 0.104 |
|  | (−1.380) | (−0.190) | (−0.160) | (−0.580) | (−1.240) | (−0.850) | (−0.520) |
| Female physicians | 0.173 | 0.168 | 0.154 | −0.049 | 0.878** | 0.378 | 0.103 |
|  | (−0.880) | (−0.680) | (−0.720) | (−0.25) | (−3.050) | (−1.640) | (−0.500) |
| Age | −0.044 | 0.013 | 0.035 | 0.017 | 0.036 | 0.050 | 0.019 |
|  | (−1.84) | (−0.440) | (−1.360) | (−0.740) | (−1.270) | (−1.900) | (−0.780) |
| Income | −0.012 | −0.012 | −0.006 | 0.000 | −0.001 | −0.005 | 0.006 |
|  | (−0.64) | (−0.52) | (−0.31) | (−0.03) | (−0.04) | (−0.25) | −0.320 |
| Education | −0.059 | 0.230 | 0.185 | 0.361* | 0.217 | 0.324 | 0.196 |
|  | (−0.34) | (−1.150) | (−1.050) | (−2.200) | (−1.170) | (−1.830) | (−1.160) |

Panel B: Monetary domain

|  | Risk | Block 1 | Block 2 | Block 3 | Block 4 | Block 5 | Block 6 |
| --- | --- | --- | --- | --- | --- | --- | --- |
|  |  |  |  |  |  |  |  |
| Male general population |  |  |  |  |  |  |  |
| (reference) |  |  |  |  |  |  |  |
| Male physicians | 0.087 | −0.158 | 0.129 | 0.190 | 0.200 | 0.201 | 0.213 |
|  | (−0.580) | (−0.84) | (−0.780) | (−1.220) | (−1.060) | (−1.160) | (−1.310) |
| Female general population | 0.289 | 0.354 | 0.487* | 0.229 | 0.477 | 0.365 | 0.100 |
|  | (−1.390) | (−1.280) | (−2.050) | (−1.060) | (−1.700) | (−1.540) | (−0.480) |
| Female physicians | 0.389* | 0.096 | 0.318 | 0.445* | 0.225 | 0.490 | 0.451 |
|  | (−1.970) | (−0.360) | (−1.340) | (−1.990) | (−0.840) | (−1.860) | (−1.920) |
| Age | 0.014 | −0.056 | −0.035 | −0.003 | −0.031 | −0.034 | −0.015 |
|  | (−0.580) | (−1.89) | (−1.33) | (−0.13) | (−1.00) | (−1.23) | (−0.57) |
| Income | −0.024 | 0.005 | 0.003 | −0.022 | 0.011 | 0.012 | 0.006 |
|  | (−1.33) | (−0.220) | (−0.150) | (−1.10) | (−0.500) | (−0.570) | (−0.300) |
| A bachelor’s degree or higher | −0.024 | 0.213 | 0.248 | 0.264 | 0.048 | 0.231 | 0.056 |
|  | (−0.14) | (−1.010) | (−1.360) | (−1.520) | (−0.230) | (−1.240) | (−0.320) |

Note: t-statistics in parentheses. * p < 0.05, ** p < 0.01, *** p < 0.001

**Appendix D: Sensitivity analysis without excluding switching back respondents**

Panel A: Health domain

|  | Risk | Block 1 | Block 2 | Block 3 | Block 4 | Block 5 | Block 6 |
| --- | --- | --- | --- | --- | --- | --- | --- |
| Male general population |  |  |  |  |  |  |  |
| (reference) |  |  |  |  |  |  |  |
| Male physicians | 0.459 | 0.326* | 0.322 | 0.526** | 0.481** | 0.485** | 0.543** |
|  | (-1.34) | (-2) | (-1.71) | (-2.64) | (-2.61) | (-2.59) | (-2.65) |
| Female general population | 0.478 | 0.0597 | 0.0136 | 0.0766 | 0.106 | 0.118 | -0.00942 |
|  | (-0.97) | (-0.26) | (-0.05) | (-0.27) | (-0.4) | (-0.44) | ((-0.03) ) |
| Female physicians | 0.716 | 0.454 | 0.396 | 0.319 | 0.960*** | 0.742** | 0.349 |
|  | (-1.47) | (-1.95) | (-1.47) | (-1.12) | (-3.63) | (-2.77) | (-1.19) |
| Age | -0.0237 | 0.0176 | 0.0272 | 0.0265 | 0.0537 | 0.0787* | 0.0302 |
|  | (-0.41) | (-0.63) | (-0.84) | (-0.78) | (-1.71) | (-2.47) | (-0.86) |
| Income | 0.0011 | 0.00631 | 0.0165 | 0.0137 | 0.0198 | 0.00164 | 0.0244 |
|  | (-0.02) | (-0.29) | (-0.67) | (-0.52) | (-0.82) | (-0.07) | (-0.91) |

Panel B: Monetary domain

|  | Risk | Block 1 | Block 2 | Block 3 | Block 4 | Block 5 | Block 6 |
| --- | --- | --- | --- | --- | --- | --- | --- |
| Male general population |  |  |  |  |  |  |  |
| (reference) |  |  |  |  |  |  |  |
| Male physicians | 0.363 | -0.007 | 0.254 | 0.376* | 0.221 | 0.213 | 0.239 |
|  | (-1.07) | ((-0.04)) | (-1.43) | (-2.03) | (-1.38) | (-1.21) | (-1.32) |
| Female general population | 0.702 | 0.405 | 0.478 | 0.209 | 0.552* | 0.325 | 0.146 |
|  | (-1.43) | (-1.73) | (-1.88) | (-0.79) | (-2.41) | (-1.28) | (-0.56) |
| Female physicians | 1.479** | 0.466* | 0.618* | 0.638* | 0.288 | 0.507* | 0.565* |
|  | (-3) | (-2) | (-2.43) | (-2.41) | (-1.26) | (-2) | (-2.18) |
| Age | 0.0544 | 0.0053 | -0.0331 | 0.0079 | 0.00513 | 0.00999 | -0.0102 |
|  | (-0.93) | (-0.19) | ((-1.09)) | (-0.25) | (-0.19) | (-0.33) | ((-0.33) ) |
| Income | -0.0581 | 0.0326 | 0.012 | -0.0157 | 0.0242 | 0.0248 | 0.0155 |
|  | ((-1.29)) | (-1.52) | (-0.51) | ((-0.64)) | (-1.15) | (-1.07) | (-0.65) |

Note: t-statistics in parentheses. * p < 0.05, ** p < 0.01, *** p < 0.001
